# Choline and Choline-Related Metabolites in Pediatric Short Bowel Syndrome

**DOI:** 10.3390/nu18101553

**Published:** 2026-05-14

**Authors:** Johannes Hilberath, Anna Shunova, Lena Heister, Christian F. Poets, Wolfgang Bernhard

**Affiliations:** 1 Pediatric Gastroenterology and Hepatology, Department of Pediatric Hematology and Oncology, University Children’s Hospital Tübingen, Faculty of Medicine, Eberhard-Karls-University, 72076 Tübingen, Germany; lena.heister@med.uni-tuebingen.de; 2 Department of Neonatology, University Children’s Hospital Tübingen, Faculty of Medicine, Eberhard-Karls-University, 72076 Tübingen, Germany; anna.shunova@med.uni-tuebingen.de (A.S.); christian-f.poets@med.uni-tuebingen.de (C.F.P.); wolfgang.bernhard@med.uni-tuebingen.de (W.B.)

**Keywords:** choline deficiency, betaine, enterohepatic cycle, lipoproteins, phospholipids, short bowel syndrome, intestinal failure, IFALD, SIBO, hepatic steatosis, TMAO

## Abstract

**Background**: Choline is an essential nutrient crucial for liver function. It is required for bile and lipoprotein secretion and the synthesis of both phosphatidylcholine (PC) to ensure tissue homeostasis and betaine as a methyl donor. Choline deficiency has been implicated in the pathogenesis of intestinal failure-associated liver disease (IFALD), with the strongest evidence for its contribution to hepatic steatosis in patients with short bowel syndrome (SBS). Contributing factors are (1) an impaired recycling of choline from bile PC, leading to fecal choline losses; (2) small bowel bacterial overgrowth resulting in choline degradation prior to absorption; and (3) parenteral nutrition (PN) insufficient to meet choline requirements. However, data on choline status and its metabolites in pediatric patients with SBS are scarce. **Objective:** To investigate plasma levels of choline and choline-related metabolites in children with SBS and evaluate differences according to PN dependency and the presence of hepatic steatosis. **Methods**: Retrospective analysis of data from SBS patients managed at our intestinal rehabilitation program between March 2021 and July 2025. Target parameters in plasma samples were measured using tandem mass spectrometry. Statistical analysis and group comparison of laboratory and clinical data were performed. **Results**: A total of 127 samples from 80 children with SBS (0.2–17.9 years) were analyzed. Plasma choline, betaine, and PC concentrations were low, with 25% of patients showing markedly reduced choline and betaine levels below 6.4 µmol/L and 16 µmol/L, respectively. TMAO concentrations, indicating bacterial choline degradation, showed extreme variability (0–30 µmol; normal < 3 µmol/L), being inversely correlated with plasma choline levels. PC subgroups containing eicosapentaenoic acid and docosahexaenoic acid were increased in patients receiving PN. However, the only difference between steatotic and non-steatotic patients was the decreased plasma concentrations of both choline and betaine. **Conclusions**: Patients with SBS, with and without PN, are at risk of choline and betaine deficiency, which is associated with IFALD-steatosis. Controlled trials on choline supplementation in pediatric patients with SBS are warranted.

## 1. Introduction

Pediatric short bowel syndrome (SBS) is a rare disorder caused by congenital abnormalities or extensive surgical small intestinal resection and represents the leading cause of intestinal failure (IF) in children [1,2]. Due to the critical reduction in mucosal surface, patients suffer from malabsorption of fluids, macro- and micronutrients, requiring long-term parenteral nutrition (PN) [3,4]. Length and anatomical configuration of the remaining bowel are key determinants of clinical outcome, including degree of malabsorption and PN dependence [5]. Moreover, loss of the ileo-cecal valve facilitates bacterial colonization of the small intestine and may lead to small intestinal bacterial overgrowth (SIBO) [6,7].

Despite advances in the intestinal rehabilitation management, intestinal failure-associated liver disease (IFALD) represents a relevant and potentially progressive complication [8]. Clinical manifestations range from inflammation and cholestasis to hepatic steatosis and fibrosis [8,9].

Choline is an essential nutrient, as recognized by the U.S. Institute of Medicine and the European Food Safety Authority [10,11], with key roles in liver, lipid, and homocysteine metabolism [12]. It is required for the synthesis of phosphatidylcholine (PC) and the export of triglycerides via very-low-density lipoproteins (VLDLs) [13]. In addition, choline serves as a precursor for betaine through one-carbon metabolism. For this purpose, 40% of exogenous choline is oxidized to betaine, and their plasma concentrations are directly correlated, whereas there is an inverse correlation between trimethylamine (TMA) oxide (TMAO) and these compounds [14,15].

As cellular choline uptake is proportional to plasma concentrations [16], reduced plasma choline may limit cellular availability and impair hepatic triglyceride export, thereby potentially promoting hepatic steatosis (reviewed in [17]).

It has been repeatedly highlighted that PN formulations provide insufficient choline to meet physiological requirements, despite containing PC as an emulsifier [17,18,19,20,21,22]. In adults on PN, choline deficiency has been linked to the development of hepatic steatosis [9]. Notably, low plasma choline levels are prevalent in adult patients receiving PN [23]. Normalization of plasma choline levels via continuous intravenous administration of choline chloride was associated with the resolution of steatosis from 4 weeks onwards and relapse 10 weeks after discontinuation of intravenous choline in adults [20]. A small study of 21 children on home parenteral nutrition reported an increased risk for the development of choline deficiency [24].

Choline requirements may be increased when enterohepatic circulation, cleavage, and reabsorption of biliary PC are impaired, leading to fecal choline loss, as demonstrated in cystic fibrosis (CF) with exocrine pancreatic insufficiency [14,25]. The enterohepatic cycle of PC depends on the cleavage of biliary PC by pancreatic phospholipase A2 IB (sPLA2IB) and brush-border phospholipase B (PLB), both of which are bile salt dependent. PLB is highly expressed in the distal small intestine, a bowel segment frequently lacking in patients with SBS. The choline moiety of PC is absorbed as lyso-PC, which is either reacylated and incorporated into chylomicron PC, contributing to plasma PC levels, or further cleaved to free choline, secreted into the portal vein, and extensively cleared by the liver [26]. Evidence from patients with CF suggests that disruption of this pathway may contribute to malabsorption of biliary PC and choline loss. In CF, exocrine pancreatic insufficiency with reduced sPLA2IB activity, low intestinal pH due to impaired bicarbonate secretion, altered intestinal transit, mucostasis, and SIBO may impair PC cleavage to lyso-PC, resulting in increased fecal choline loss or bacterial degradation of choline to TMA, which is oxidized to TMAO [14,27].

Moreover, impaired enterohepatic circulation and bile acid homeostasis may further compromise bile function, lipid digestion, and liver health [15,28].

Mechanisms of choline deficiency in SBS patients may include (1) fecal choline loss due to impaired (re)absorption in the absence or partial loss of the terminal ileum, (2) increased bacterial degradation in SIBO with TMAO formation, and (3) an insufficient amount of choline in PN formulations. However, systematic data on choline status in pediatric SBS patients, irrespective of PN requirement, are lacking.

Therefore, the aim of this study was to systematically assess plasma choline and choline-related metabolites in pediatric patients with SBS and to evaluate their association with PN dependency and the presence of hepatic involvement.

## 2. Materials and Methods

### 2.1. Study Population

**Figure 1:**
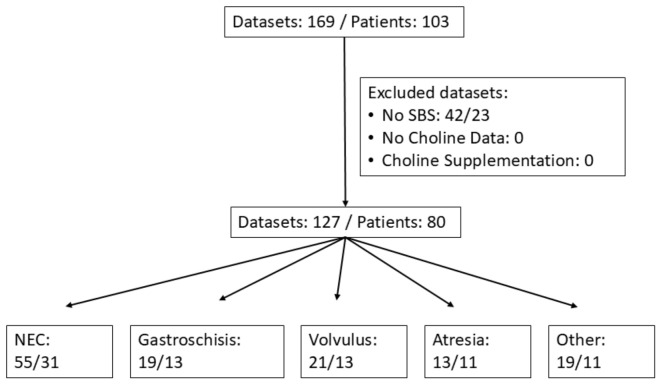
Flow diagram of study patients. The datasets included 169 from 103 patients, of which those without SBS (42 datasets/23 patients) were excluded. For multiple determinations of individual samples, the medians were calculated. Abbreviations: NEC, necrotizing enterocolitis; SBS, short bowel syndrome.

The routine assessment of plasma choline, choline metabolites, and the fatty acid composition of choline-containing phospholipids (phosphatidylcholine, PC) in children aged 0–17 years with IF was introduced at our intestinal rehabilitation center in 2021. Between March 2021 and July 2025, a total of 169 individual samples were collected from 103 patients (Figure 1).

Of these, 127 samples from 80 patients aged 0.2–17.9 years met the inclusion criteria for SBS with or without PN. There were 1–5 individual samples from separate visits. In case of multiple samples of a single patient, medians were formed from all data. Exclusion criteria were known primary liver disease, acute liver injury, documented choline supplementation, missing plasma choline measurements, and age > 18 years at the time of study inclusion. Patients receiving oral formula supplementation in addition to regular food and PN were not excluded, as both infant formulas and human breast milk contain choline, although the choline concentration may be variable or unknown [29].

### 2.2. Clinical Parameters

Clinical data were extracted from the hospital’s software (SAP Version 2020 and 2021, SAP SE & Co. KG, Walldorf, Germany). PN dependency was quantified using the parenteral nutrition dependency index (PNDI), calculated as the ratio of non-protein energy intake (NPEI) to resting energy expenditure (REE, estimated using the Schofield Equation 1985 [30]: PNDI [%] = (NPEI/REE) × 100. Parenteral lipid supply was documented as SMOFlipid alone in 55 patients, Omegaven alone in 1 patient, a combination of both in 6 patients, and no parenteral lipid supply in 2 patients. Both lipid emulsions were supplied by Fresenius Kabi Deutschland GmbH, Homburg, Germany.

### 2.3. Plasma Collection

EDTA blood (1.0–2.7 mL) was obtained by venipuncture, kept on crushed ice, and centrifuged within 1 h at 1000× *g* for 10 min at room temperature. Plasma supernatant was immediately aspirated and aliquoted into 100 µL samples, frozen at −20 °C, and transferred to a −80 °C freezer within 5 days until analysis.

### 2.4. Measurement of Choline and Its Metabolites

Patients were routinely screened for plasma concentrations of choline, betaine, dimethylglycine (DMG), trimethylamine oxide (TMAO), phosphatidylcholine (PC), lyso-PC, sphingomyelin (SPH), and carnitine. PC was further differentiated into subclasses according to their fatty acid content, that is, comprising either two saturated fatty acids (Sat.-PC) or an oleic acid (OA) (C18:1-PC), linoleic acid (LA) (C18:2-PC), arachidonic acid (ARA) (C20:4-PC), eicosapentaenoic acid (EPA) (C20:5-PC), or docosahexaenoic acid (DHA) residue (C22:6-PC), together with a saturated fatty acid (mostly palmitic or stearic acid) [14,31].

### 2.5. Mass Spectrometry

Plasma samples were processed using standard procedures, as previously described [14,31]. Briefly, 50 µL of plasma was spiked with an internal standard (D4-choline chloride) and extracted with chloroform:methanol, as described by Bligh & Dyer [32]. The upper water–methanol phase, containing the water-soluble metabolites (choline, betaine, TMAO, and others), was separated from the lipid-containing chloroform phase. Diarachidoyl–PC (PC20:0/20:0) was added as an internal mass spectrometry standard after phase separation to an aliquot of the chloroform extract, as Bligh & Dyer extraction is quantitative [32], to obtain sample aliquots without additional arachidic acid for potential gas chromatography.

The equipment used for the analysis comprised a TSQ Quantum Discovery Ultra tandem mass spectrometer, Finnigan Surveyor Autosampler Plus, and Finnigan Surveyor MS Pump Plus (Thermo Fisher Scientific, Dreieich, Germany). Choline, D4-choline, betaine, and TMAO were separated on a Polaris Si-A^®^ column (2 × 100 mm inner diameter; 3.0 µm particle size; Agilent Technologies, Santa Clara, CA, USA) at 40 °C. Elution was performed at 0.5 mL/min with solvent A (methanol:water:formic acid; 95:5:1; *v*/*v*) and B (water:formic acid, 99:1; *v*/*v*): Gradient conditions were 100% A (0–2 min) → 50% B (2–4 min) → 100% A (4–5 min) → 100% A (5–7 min), and the components were analyzed at positive ionization in the selected reaction monitoring (SRM) mode, using mass by charge (*m*/*z*) transitions of +104→ +60 (choline), +108→ +60, +61 (D4-choline), +118→ +59 (betaine), +104→ +58 (dimethylglycine), and +162→ +60 (carnitine). PC, lyso-PC, and SPH were separated isocratically on a Polaris Si-A (2 × 150 mm i.d.; 2 µm; Agilent Technologies, Santa Clara, CA, USA) with chloroform:methanol:300 mM ammonium acetate (60:38:2%, *v*/*v*) as the mobile phase. Phosphorylcholine (mass/charge [*m*/*z*] = +184) served as the diagnostic fragment.

### 2.6. Statistics

Data management was performed using Microsoft Office Excel (version 2021). To eliminate unbalanced documentation of individual patients with multiple determinations (2–5) and to reduce errors due to single extremely low or high values, the medians of an individual’s data were formed. This resulted in 80 datasets of 127 samples from 1 to 5 different visits. All analytical and clinical data are expressed as medians and interquartile ranges. Significance values were determined by non-parametric testing for group comparison (Mann–Whitney U-statistic) and non-parametric correlation (Spearman’s rank correlation coefficient), using GraphPad InStat, version 3.10 (STATCON GmbH, Witzenhausen, Germany). *p*-values <0.05 were considered significant.

## 3. Results

### 3.1. Basic Data of the Study Group

Basic data of the study group are shown in the flow sheet (Figure 1). Out of 103 patients, 80 with SBS were included, who did not receive choline supplementation and were below 18 years of age.

#### 3.1.1. Biometric Characteristics and Routine Laboratory Parameters

- Biometric characteristics and routine laboratory parameters are summarized in Table 1. NEC was the most common cause of SBS (39%), followed by gastroschisis, volvulus, atresia, and other causes (including focal intestinal perforation (FIP), surgical complications, and extensive aganglionosis), each accounting for 14–16% of cases. Age ranged from 0.2 to 17.9 years (median 6.3). Median body weight was 18.8 kg (range 3.6–65.5). Eighty percent of patients (*n* = 64) received PN with a median parenteral nutrition dependency index (PNDI) of 91.2%. The residual short bowel length was 40 cm (24–78 cm). Twenty percent of patients (*n* = 16) were diagnosed with hepatic steatosis, including 14 identified by ultrasound and two confirmed by liver biopsy. Of the 14 patients with hepatomegaly, three had hepatic steatosis, and 11 did not.
- Median plasma albumin concentration and prothrombin time (Quick) were within the normal range but showed considerable variability, with minimum values of 2 g/dL and 31%, respectively. Cholesterol and triglyceride levels were generally low, although occasionally elevated values were observed. Phosphatidylcholine (PC) was mostly below the internal reference range (median 1.71 mmol/L [1.55–1.88], with some values exceeding 1.88 mmol/L. Median aspartate aminotransferase (AST) and alanine aminotransferase (ALT) values were at the upper limit of the normal range, whereas gamma-glutamyltransferase (gGT) levels were normal. However, some patients showed extreme values (Table 1). No significant differences in anthropometric or other parameters were observed between SBS subgroups (gastroschisis, volvulus, atresia, other causes; see online Supplementary Table S1).

#### 3.1.2. Choline Parameters in the SBS Study Group

**Figure 2:**
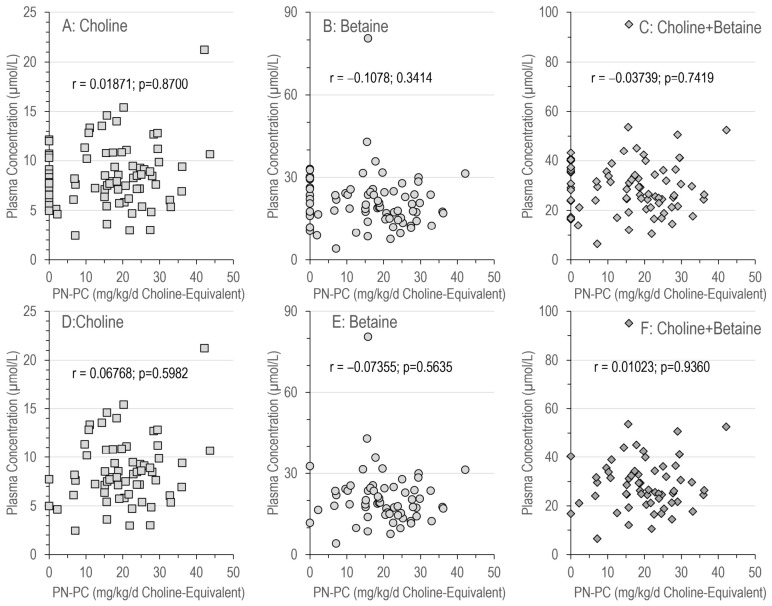
Concentrations of choline (**A**,**D**), betaine (**B**,**E**) and their sum (**C**,**F**) in relation to the amount of choline administered intravenously as PC. Parenteral PC is expressed as the amount of choline in PC (choline equivalent). (**A**–**C**) show the data of all patients, whereas (**D**–**F**) only show those individuals with parenteral nutrition. Abbreviations: PN-PC, PC administered parenterally as a choline carrier; r, coefficient of correlation; and *p*, significance level.

- Depending on the amount of PN fat administered (see Section 2—Materials and Methods), patients with PN received 16.4 [1.9–26.7] g fat per day (range: 0.0–75.7 g/d), i.e., 1.20 [0.93–1.58] g/kg/d. Accordingly, at ~1.2 g egg-PC/10 g triglyceride, the amount of parenteral PC was 2.15 [1.37–3.71] g/d. At a mean molecular weight of PC of 750 Da, and 104.17 Da for choline, this equals 299 [190.0–514.6] mg choline per day, and 20.0 [15.5–26.3] (range: 0.0–43.7) mg/kg/d of choline, delivered in the form of PC as an emulsifier.
- There was no correlation between the amount of parenteral PC administration and the plasma concentrations of choline, betaine and their sum (Figure 2), neither for the whole patient group (Figure 2A–C) nor for those with PN (Figure 2D–F).

- Table 2 shows the overall values of choline-related parameters in SBS patients relative to the reference values. While there was no significant difference in the entire group for choline and TMAO (Table 2A), choline values were occasionally only 2.5 µmol/L, whereas TMAO reached 32 µmol/L. However, the concentrations of betaine and the sum of choline and betaine, which showed a large range, were significantly decreased. Median TMAO levels differed only slightly between patients with and without an ileocecal valve. Although TMAO levels varied widely in both groups, choline-related parameters were comparable, despite differences in liver enzymes between these sub-groups (Table S3).
- Notably, 45/80 (56.3%), 62/80 (77.5%), and 45/80 (56.3%) of patients with SBS had choline, betaine, and combined choline + betaine levels below the median of controls, respectively. Furthermore, 24/80 (30%), 47/80 (58.8%), and 50/80 (62.5%) were within the lowest quartile of control values for these parameters.
- Notably, carnitine, which requires choline/betaine-derived methyl groups for synthesis, was also decreased in the entire study group, whereas dimethylglycine (DMG), the demethylation product of betaine, was not significantly changed.
- For choline and choline-related compounds in SBS subgroup patients, see Supplementary Table S2.

#### 3.1.3. Age-Related Changes in Water-Soluble Choline Compounds in Plasma

**Figure 3:**
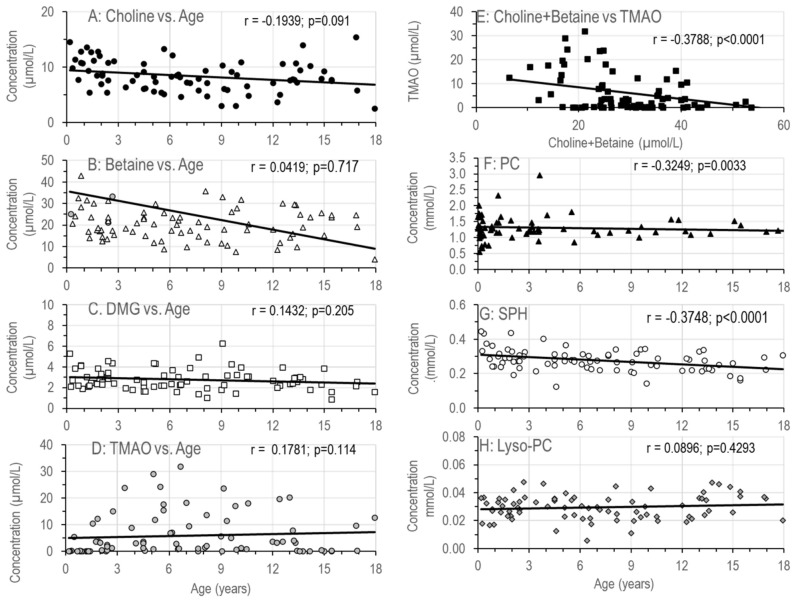
Plasma concentrations of choline (**A**), its water-soluble derivatives betaine and di-methylglycine (DMG) (**B**,**C**), and of TMAO (**D**) in relation to age. (**E**) shows the ratio between plasma TMAO and the sum of choline and betaine, while (**F**–**H**) shows plasma concentrations of the choline-containing plasma phospholipids in relation to age. Data are individual values of 80 patients aged 0.2–17.9 years with SBS, with or without parenteral nutrition. Abbreviations: DMG, dimethylglycine; lyso-PC, lyso-phosphatidylcholine; PC, phosphatidylcholine; r, Spearman coefficient of variation; *p*, significance value; SPH, sphingomyelin; TMAO, trimethylamine oxide.

- Figure 3A–D shows the median individual concentrations of plasma choline and its major water-soluble derivatives in relation to age. There were no age-dependent changes in plasma choline or DMG levels, whereas betaine levels decreased. Notably, betaine levels showed a large range (Figure 3B). Consequently, the sum of choline and betaine (Figure 3A,B), representing a measure of choline/methyl group pools, decreased with age.

- TMAO, as a parameter of intestinal bacterial choline/betaine degradation, showed no age dependency. Whereas the median value was not different from controls, TMAO levels in SBS patients showed a large range from near zero up to 32 µmol/L (Figure 3D).
- An inverse relationship was observed between plasma TMAO levels and those of choline and betaine (Figure 3E).
- Figure 3F–H shows the plasma concentrations of choline-containing phospholipids with a small but significant decrease with age for PC and SPH, but not for lyso-PC.
- Carnitine levels were decreased compared to control levels (Table 2), but did not correlate with age (r = −0.0754; *p* = 0.506).

#### 3.1.4. Phospholipids Containing a Choline Headgroup

**Figure 4:**
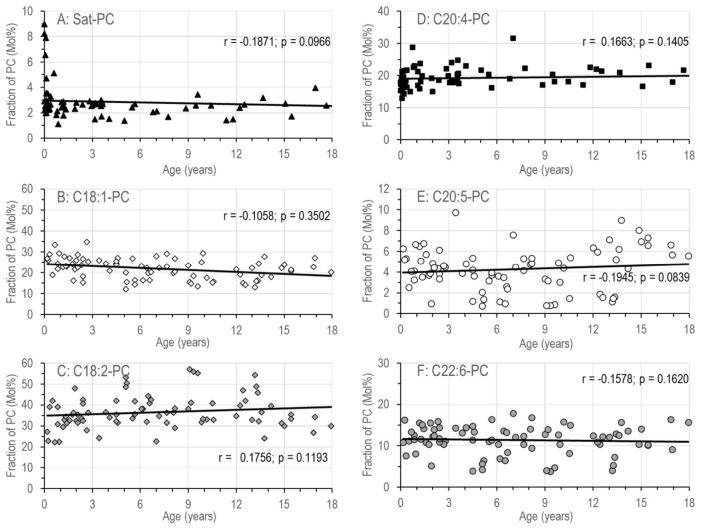
Molar fractions of individual PC subgroups versus age. PC molecular species were analyzed and grouped into those comprising only saturated fatty acids (Sat-PC) (**A**) or a saturated fatty acid together with an unsaturated fatty acid, namely oleic acid (C18:1-PC) (**B**) or linoleic acid (C18:2-PC) (**C**). D-F show those PC sub-groups comprising a long-chain polyunsaturated fatty acid residue, like arachidonic acid (C20:4n-6), eicosapentaenoic acid (C20:5n-3) or docosahexaenoic acid (C22:6n-3), i.e., C20:4-PC (**D**), C20:5-PC (**E**) or C22:6-PC (**F**), respectively. Data are single data points of median values of 80 SBS patients. Abbreviations: PC, phosphatidylcholine; C18:1, oleic acid; C18:2, linoleic acid; C20:4, arachidonic acid; C20:5, eicosapentaenoic acid (EPA); C22:6, docosahexaenoic acid, (DHA); Sat, saturated; r, Spearman coefficient of variation; and *p*, significance value.

- All phospholipids containing a choline headgroup were decreased, i.e., PC, SPH, and lyso-PC (Table 2B), and the fractions of PC-subgroups were significantly altered (Table 2C): PC comprising two saturated fatty acids (Sat.-PC) or an oleic acid (OA, C18:1n-9) residue (C18:1-PC) were increased. This also applied to PC compounds containing an omega-3 long-chain polyunsaturated fatty acid (LC-PUFA) residue, i.e., eicosapentaenoic (C20:5n-3) or docosahexaenoic acid (C22:6n-3). These compounds (C22:6-PC, C20:5-PC) were increased at the expense of PC coupled to omega-6-fatty acids, i.e., C18:2-PC and C20:4-PC (Table 2C).
- Phospholipid classes and PC subgroups did not show significant changes between the ages of 0.2 and 17.8 years. This applied to those being fully saturated or comprising an OA or LA residue (Figure 4A–C), as well as to those containing a LC-PUFA residue, i.e., ARA, EPA or DHA (C20:4-PC, C20:5-PC, C22:6-PC) (Figure 4D–F).

#### 3.1.5. Parameters of Choline Homeostasis in SBS Sub-Groups

**Figure 5:**
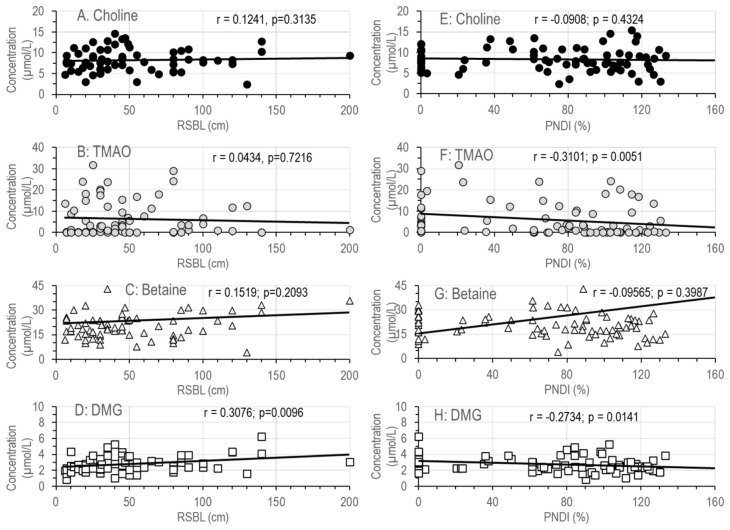
Concentrations of choline (**A**,**B**), the downstream metabolites of choline, betaine (**C**,**D**) and dimethylglycine (**E**,**F**), and TMAO (**G**,**H**) in relation to residual small bowel length (**A**,**C**,**E**,**G**) or parenteral nutrition dependency index (**B**,**D**,**F**,**H**). Data are single data points of the median values of 80 SBS patients. Abbreviations: DMG, dimethylglycine; PNDI, parenteral nutrition dependency index; RSBL, residual small bowel length; TMAO, trimethylamine oxide; r, coefficient of correlation; *p*, significance level.

- No correlation was observed between residual small intestinal bowel length (RSBL) or parenteral nutrition dependency index (PNDI) and choline or betaine levels (Figure 5A–D) in these patients. In particular, the concentrations of betaine showed considerable variability, ranging from normal values (>30 µmol/L) to below 10 µmol/L (Figure 5C,D). Concentrations of dimethylglycine (DMG), the demethylation product of betaine during the synthesis of methionine from homocysteine, were directly proportional to RSBL and inversely correlated with PNDI (Figure 5E,F). TMAO levels ranged from below the detection level (<1 µmol/L) up to 32 µmol/L (Figure 5G,H), and were inversely related to the PNDI (Figure 5H).

#### 3.1.6. No Parenteral vs. Parenteral Nutrition

- Direct comparison of patients without and those receiving PN revealed only minor but statistically significant differences, including slightly higher liver enzymes and lower cholesterol and triglyceride levels in the PN group (Table 3A). There were no significant differences in plasma choline levels or its major water- or lipid-soluble metabolites (Table 3B). In contrast, carnitine and dimethylglycine (DMG) levels were decreased in PN patients. TMAO was highly variable, both in patients without PN (3.8 [2.8–8.6] µmol/L) as well as with PN (1.3 [0.1–9.5] µmol/L), with no significant differences. Similarly, phospholipid classes showed no differences (Table 3C), but PC subgroups comprising an LA (C18:2-PC) or ARA residue (C20:4-PC) were decreased, whereas those comprising an EPA or DHA residue (C20:5-PC, C22:6-PC) were increased (Table 3D). Notably, there was no correlation between the amount of parenterally given choline in its lipid-bound form (PN-PC) and the plasma concentrations of choline, betaine and their sum (Figure S1).

#### 3.1.7. Presence or Absence of the Ileocecal Valve

- Differences in clinical and choline-related parameters between patients with and without an intact ileocecal valve are presented in Table S3. Plasma choline, betaine, DMG, choline-containing phospholipids, and PC subgroups were comparable between groups. TMAO levels were lower in patients without an intact ileocecal valve.

#### 3.1.8. Non-Steatotic vs. Steatotic Patients

- When comparing patients with or without hepatic steatosis (Table 4), the data showed no differences in age distribution or body mass index (BMI). RSBL was higher and PNDI lower in non-steatotic patients, but liver enzymes, prothrombin time or conjugated bilirubin showed no difference between groups (Table 4A). Cholesterol concentration was lower in the steatosis group. Notably, patients with steatosis received higher parenteral amounts of both triglycerides and esterified choline (PC), despite having lower plasma choline and betaine concentrations (Table 4). No characteristic sex or age-specific differences were observed (Supplementary Figure S1).
- Table 4B shows that TMAO levels were identical in both the non-steatotic and steatotic groups, with normal median levels but extreme values of up to 32 and 24 µmol/L, respectively. However, in contrast to the comparison of patients with PN versus those without PN, choline, its direct metabolite betaine, and the sum of choline and betaine as an established parameter of long-term choline status [33], were significantly decreased in SBS patients with hepatic steatosis.
- Although carnitine levels were lower in patients with PN than in PN-weaned individuals (Table 3), there was no difference in those with and without steatosis, showing an extreme range in either group (Table 4).

## 4. Discussion

This retrospective observational study provides a comprehensive evaluation of choline status and its related metabolites in children with short bowel syndrome. With over 100 measurements in 80 pediatric SBS patients, it represents the largest cohort reported to date. To the best of our knowledge, this is the first study to assess choline status in both patients with SBS receiving (partial) parenteral nutrition and those fully weaned to enteral feeding.

Plasma choline concentrations were below the reference range in 56% of children. Severe choline deficiency, reflected by choline and betaine levels below 6.4 µmol/L and 16 µmol/L, respectively, was found in 25% of patients. In 1999, Misra et al. reported an increased risk of choline deficiency in 21 children receiving long-term PN for various underlying conditions [24]. Notably, in our study, choline status did not differ between patients receiving PN and those who had achieved enteral autonomy, suggesting that the risk of choline deficiency extends beyond PN dependence in SBS patients. This parallels observations for other micronutrients, where deficiencies can persist despite transition to enteral nutrition in children with intestinal failure [3,34].

Although its addition has been advocated for more than a decade, parenteral nutrition fails to meet physiological choline requirements, reflecting the limited provision of water-soluble/free choline in standard formulations [21,22]. Although PN lipid emulsions supplied phospholipid-bound choline as egg-PC emulsifiers, parenteral PC-bound choline intake was not associated with plasma choline, betaine, or their sum. This observation supports the distinction between lipid-bound choline administered parenterally and water-soluble/free choline, suggesting that parenteral PC does not effectively compensate for impaired plasma choline status in SBS. Interestingly, we observed no statistically significant correlations between choline and betaine status and residual small bowel length or the degree of PN dependency. Nevertheless, choline deficiency was also prevalent in patients weaned off parenteral support, indicating that factors beyond PN contribute to low choline status in this population. Potential mechanisms include (1) insufficient oral/enteral supply, (2) disrupted reabsorption of choline from biliary PC following extensive surgical resections, and/or (3) bacterial degradation of choline within the small intestine.

Although small intestinal bacterial overgrowth (SIBO) was not systematically assessed in our cohort, it is prevalent in children with intestinal failure [35]. Bacterial degradation of choline, reflected by TMAO formation, provides indirect evidence of this pathway. This is supported by the significant inverse correlation between plasma TMAO levels and choline and betaine observed in our study. Interestingly, high TMAO levels could not simply be attributed to the absence of the ileocecal valve; in fact, TMAO levels were lower in patients without an intact ileocecal valve. We can only speculate on the contributing factors, which may include differences in SIBO prevalence, intestinal microbiota composition, stasis, overall motility, or altered intestinal transit time due to loss of the regulatory function of the ileocecal valve. Nevertheless, choline degradation may occur either within the small intestine before absorption or in the colon from non-absorbed choline or lyso-PC [25].

Whole-body choline turnover is relatively low, accounting for only about 0.5% of the total choline pool (approximately 400–550 mg/d in adults [10,11]). In contrast, hepatic PC turnover via biliary secretion and subsequent enteral reuptake is substantial, corresponding to approximately 50% of the hepatic PC pool per day, which is approximately 11 g/day of PC, or about 1500 mg/d of choline, which is threefold higher than the adequate intake [36]. Animal studies have shown that enterohepatic recycling of bile PC may exceed 50% of the hepatic PC pool per day, and that biliary PC secretion is maintained even when hepatic PC biosynthesis is impaired under choline-deficient conditions [37]. Consequently, disruption of the enterohepatic circulation following ileal resection may lead to increased fecal losses of PC. However, fecal or stomal losses were not quantified in the present study.

Choline deficiency may be further aggravated by frequent single-nucleotide polymorphisms (SNPs) of the phosphatidylethanolamine (PE)-N-methyltransferase (PEMT) gene. While the PEMT pathway is insufficient to compensate for exogenous choline needs, its deficiency further increases exogenous choline requirements, and the combination of fecal loss, SIBO, and the frequent PEMT-SNP rs 12325817 leads to extremely low plasma choline levels [14,27,37].

The methyl group of betaine is either directly transferred to homocysteine, resulting in methionine that is activated to S-adenosylmethionine (SAM), or further downstream metabolites of betaine (dimethylglycine [DMG] and sarcosine) serve as methyl/one-carbon donors to convert homocysteine to methionine under the participation of tetrahydrofolate and vitamin B12 [14,27]. SAM acts as a methyl donor for creatine synthesis, histone, and DNA methylation (epigenetics), and the PEMT pathway (as described above). Another function of SAM is to stimulate the hepatic transsulfuration pathway, converting homocysteine to cysteine and further on glutathione (GSH) formation, which is defective in choline-deficient CF patients [27,28,38,39,40]. Hence, choline is key to hepatic processes beyond the formation of membranes, bile, and lipoproteins.

Choline is an essential nutrient for humans, particularly in children, where physiologic plasma choline levels and requirements are higher than in adults [10,11]. Choline deficiency, as indicated by low plasma choline concentrations, has been linked to the pathogenesis of intestinal failure-associated liver disease, particularly hepatic steatosis in adult patients [26]. Available evidence suggests that hepatic steatosis is negatively correlated with plasma choline concentrations [14,19,20,27,41].

Our data extend these observations to children with short bowel syndrome. We found a significant association between an impaired choline status and the presence of hepatic steatosis, both in PN-dependent children and in those being weaned off from PN. These findings need to be interpreted in the context of substantial improvements in the care of pediatric SBS over the last decades: management by multidisciplinary intestinal rehabilitation teams, including optimized PN strategies and the use of composite lipid emulsions, has markedly reduced the incidence and mortality of progressive IFALD requiring transplantation [42]. In line with this, patients in our cohort largely exhibited preserved liver function, as reflected by median bilirubin levels and prothrombin time within the reference range, and only mild elevations in transaminases, gGT and AP, indicating a high standard of care within our intestinal rehabilitation program. Notably, however, hepatic steatosis was diagnosed in 17.5% of patients using ultrasound or liver biopsy. Higher prevalences have been reported by Mutanen et al., with steatosis present in 50% of PN-dependent and 45% of weaned pediatric intestinal failure patients, suggesting that steatosis may persist even after discontinuation of PN [43]. Therefore, it is important to emphasize our finding that choline deficiency was not restricted to PN-dependent patients but was similarly prevalent in those weaned off PN. Beyond its high prevalence, the clinical relevance of hepatic steatosis in IFALD is underscored by its potential progression to fibrosis and hepatic failure [9]. Established contributors to IFALD are categorized in patient- and PN-related factors [44]: prematurity, small intestinal bacterial overgrowth, sepsis episodes, interrupted enterohepatic circulation, and composition, amount and duration of PN, including lack of antioxidants and micronutrient imbalances. Within this multifaceted framework, choline deficiency should therefore not be viewed as an isolated driver of IFALD, but as one potentially modifiable factor. Our data suggests that impaired choline and betaine status may represent a currently under-recognized mechanism contributing specifically to hepatic steatosis in children with otherwise controlled disease.

Previous studies by Buchman et al. described an association between choline deficiency and hepatic steatosis in patients with SBS and other PN-dependent patients [18,19,20]. Our analysis of downstream metabolites extends these findings and suggests a complex metabolic interplay. Both PN-treated and PN-independent SBS patients showed a wide range of plasma choline and betaine levels, as well as their combined levels. A similar variability was observed for plasma phospholipids. However, it remains unclear to what extent low plasma phospholipids indicate impaired hepatic function or reduced hepatocyte mass. In our cohort, plasma PC concentrations correlated not only with choline but also with biochemical markers of hepatic injury (AST, ALT, and gGT), whereas no correlation was found with plasma betaine. The reasons for these correlations and their clinical significance require further investigation. This is particularly relevant because plasma PC—whether of endogenous formation or derived from PN—does not appear to prevent hepatic steatosis in the setting of choline deficiency, as previously reported in CF patients [27,41,45].

In adults, parenteral administration of choline chloride in addition to PN formulations—currently devoid of free choline, despite being recommended [21,22]—restored plasma choline levels and resolved steatosis within 3 months [19]. The rapid recurrence of hepatic steatosis after discontinuation of choline supplementation in adults highlights the need for continued choline administration [19,20]. This similarly applies to patients with choline-deficient cystic fibrosis being treated with enteral choline administration [27,41]. Hence, while choline supplementation has been shown to be potentially effective and safe in small trials in adults and in patients with cystic fibrosis, prospective randomized trials are required to establish its role in pediatric SBS patients with long-term parenteral nutrition. In SBS, parenteral choline supplementation may be preferable, as bacterial choline degradation in the small intestine and loss of the terminal ileum may limit efficient enteral choline absorption.

Of note, instead of investigating plasma choline alone, we also analyzed its water-soluble downstream metabolites betaine, dimethylglycine (DMG), trimethylamine oxide (TMAO), as well as phosphatidylcholine (PC), sphingomyelin (SPH), and lyso-PC. Betaine, together with choline, is considered a useful marker of choline status, as a significant fraction of administered choline is rapidly converted to betaine [27,41]. DMG is formed when a methyl group is transferred to homocysteine, resulting in methionine. Hence, choline plays a major role as a methyl/one-carbon donor for methionine synthesis, whereas carnitine synthesis requires activated methionine (S-adenosylmethionine = SAM) as a methyl source. TMAO is an indicator of bacterial choline degradation prior to its absorption, and is associated with dysfunction of the enterohepatic cycle/reabsorption of bile PC in patients with CF [14,27]. Finally, choline-containing phospholipids, namely PC, SPH, and lyso-PC, were decreased, suggesting impaired metabolism of phospholipids containing a choline head group. PC represents the equilibrium of lipoproteins, comprising 20–50% PC in their lipid fraction, whereas SPH is mainly present in the plasma membrane and an increase may indicate cellular damage [13].

Despite the different SBS causes, there were no group differences in choline or other parameters. However, in general, choline, betaine, and, particularly, TMAO showed great variability across patients, with choline and betaine partly being extremely low (<2.5 µmol/L and 4 µmol/L, respectively). This also applied to PC and SPH, confirming previous data on low plasma phospholipids in CF patients with disturbed enterohepatic choline circulation [14,27,41,45]. Lyso-PC, as a potential indicator of inflammation, however, was low in all patients [46]. Further investigation is required to determine whether other metabolic similarities exist between CF and SBS patients, particularly for parameters related to choline/betaine metabolism, namely methionine, homocysteine, glutathione, taurine, and others.

Choline is critical for hepatic lipoprotein metabolism and bile secretion, as well as for methyl group donation via betaine, which is involved in homocysteine degradation, epigenetic control, and other methylation reactions [12,14]. Notably, hepatic choline metabolism is functionally linked to that of extrahepatic organs, including the lungs, through the PC moiety of HDL and VLDL [47,48,49]. Total choline concentrations (i.e., PC, lyso-PC, sphingomyelin (SPH), and water-soluble choline components) are tightly regulated in all organs and are highest in the liver, followed by the lungs and brain [50].

Therefore, potential benefits of maintaining an adequate choline status may extend beyond IFALD-steatosis. Although these aspects were not addressed in the present study, choline deficiency has been linked to skeletal muscle abnormalities, impaired lung function, renal damage, an increased risk for venous catheter thrombosis, and adverse neurological and developmental effects [27,51,52,53]. Taken together, these potential hepatic and non-hepatic consequences raise the question of how choline monitoring and supplementation could be integrated into current intestinal rehabilitation strategies. Therefore, studies exploring the effects of (intravenous) choline supplementation in children with intestinal failure are warranted.

This study has several limitations. First, its retrospective design and reliance on chart review, including ultrasound and biopsy data for hepatic fat assessment, limit the ability to establish causal relationships. However, laboratory and nutritional data were obtained at the time of study enrollment. Second, the study lacked a healthy control group, and reference values for choline and its metabolites were based on measurements established in our laboratory, as no universally established pediatric reference intervals are currently available. Nevertheless, these reference values align with published data, and the absence of relevant age-related correlations for most choline-related parameters in SBS patients supports their use across age groups. Moreover, although sex and age distributions were comparable, subgrouping resulted in very small group sizes, limiting the robustness of sex- and age-specific analyses. Third, SIBO, fecal or stomal choline losses, and enteral choline intake were not assessed, restricting mechanistic insights into the discussed pathophysiology of choline deficiency in pediatric SBS patients. Finally, PEMT single-nucleotide polymorphisms that may influence individual choline metabolism and status were not assessed. Future prospective studies are needed to address these limitations.

## 5. Conclusions

Low plasma concentrations of choline and betaine, accompanied by elevated TMAO levels, are common in pediatric patients with SBS, irrespective of PN dependency. Our findings identify pediatric SBS patients as a population at risk for impaired choline status and demonstrate an association between reduced plasma choline and betaine levels and the presence of hepatic steatosis. To the best of our knowledge, this study provides the first systematic characterization of choline status and its metabolism in a clinically relevant cohort of children with SBS. These findings support consideration of routine assessment of choline status in children with SBS. Within the multifactorial pathophysiology of IFALD, choline deficiency may represent a modifiable contributor, particularly to hepatic steatosis. Prospective studies are needed to confirm our findings. Furthermore, while choline supplementation has shown beneficial effects in small trials in adults receiving PN, controlled studies are required to establish its efficacy, safety, and clinical benefits in pediatric SBS.

## Figures and Tables

**Table 1:** Characteristics of study patients with short bowel syndrome (SBS).

Number of patients (and datasets)	80 (127)	
Sex (male/female) (*n*; %)	44/36 (55%/45%)	
SBS etiology:	31 (38.8)	
NEC [*n*, (%)]	13 (16.3)	
Gastroschisis [*n*, (%)]	13 (16.3)	
Volvulus [*n*, (%)]	11 (13.8)	
Atresia [*n*, (%)]	12 (15)	
Age at study inclusion (years)	6.3 (2.4–10.5) [0.2–17.9]	
Sex (male/female)	44/36 (55%/45%)	
Body weight (kg)	18.8 (11.3–27.2) [3.6–65.5]	
Body length (cm)	112.5 (86.3–132.2) [50.5–169.0]	
BMI (kg/m^2^)	15.5 (14.3–16.5) [11.4–26.2]	
Parenteral nutrition (no/yes)	16/64 (20%/80%)	
If yes: Parenteral fat supply (g/kg/d)	1.2 (0.9–1.6) [0.0–2.6]	
Parenteral choline as PC (mg/kg/d)	20.0 (15.4–26.2) [0.0–43.7]	
Formula (no/yes/unknown)	30/46/4 (37.5%/57.5%/5%)	
Stoma (No/Yes)	68/12 (85%/15%)	
ICV present (no/yes/unknown)	59/20/1 (37.75%/25%/1.25%)	
RSBL (cm)	40.0 (24–78) [6–200]	
PNDI (%) [*n* = 62]	91.2 (68.5–112.1) [3.3–160.2]	
Hepatomegaly (No/Yes)	64/16 (80%/20%)	
Hepatic steatosis (No/Yes)	66/14 (82.5%/17.5%)	
Parameter	Patients	Reference value
Albumin (g/dL)	3.5 (3.2–3.8) [2.0–4.4]	3.0–5.0
Prothrombin time (Quick)	81.0 (68.3–94.0) [31.0–120.0]	70–120
C-reactive protein (mg/dL)	0.1 (0.0–0.2) [0.0–5.7]	<0.5
Cholesterol (mg/dL)	101 (78–114) [33–220]	130–190
Triglycerides (mg/dL)	59 (42–80) [18–232]	<200
Phosphatidylcholine (mmol/L)	1.24 (1.10–1.49) [0.55–2.96]	1.71 (1.55–1.88)
AST [U/L]	40 (27–51) [18–392]	<39
ALT [U/L]	38 (24–57) [11–488]	<39
gGT [U/L]	21 (13–37) [7–427]	<30
AP [U/L]	279 (205–332.9) [103–809]	Age dependent

Biometric and serological data of SBS patients. If not otherwise stated, data are presented as medians (interquartile range) and [range] of 80 patients. Abbreviations: AST, serum aspartate aminotransferase; ALT, serum alanine aminotransferase; BMI, body mass index; FIP, focal intestinal perforation; gGT, gamma glutamyl transferase; ICV, ileocecal valve; NEC, necrotizing enterocolitis; PNDI, parenteral nutrition dependency index; RSBL, residual short bowel length; SBS, short bowel syndrome; and SIBO, small intestinal bacterial overgrowth.

**Table 2:** Choline parameters of study patients with SBS.

Parameter	SBS Patients	*p* Value	Reference Values *
A: Water-Soluble Compounds (µmol/L)			
Choline	8.2 (6.4–10.5) [2.5–15.4]	0.4885	8.6 (7.2–9.6)
Betaine	20.2 (15.5–25.6) [4.0–193.8]	**<0.0001**	26.1 (22.6–30.0)
Choline + betaine	29.4 (23.6–36.3) [6.5–219.0]	**0.0015**	34.5 (30.9–39.6)
DMG	2.5 (2.1–3.2) [0.8–6.2]	0.0549	3.0 (2.4–4.0)
TMAO	2.6 (0.2–9.5) [0.0–31.8]	0.8062	2.1 (1.1–2.9)
Carnitine	15.4 (10.4–22.8) [2.1–58.1]	**0.0007**	20.9 (18.7–27.0)
B: Choline-Containing Lipids (mmol/L)			
PC	1.24 (1.10–1.49) [0.55–2.96]	**<0.0001**	1.50 (1.35–1.78)
SPH	0.27 (0.24–0.31) [0.12–0.45]	**<0.0001**	0.36 (0.32–0.40)
Lyso-PC	0.030 (0.023–0.036) [0.006–0.048]	**<0.0001**	0.049 (0.039–0.057)
C: PC Sub-Groups (Mol-% of PC)			
Sat.-PC	2.6 (2.3–2.9) [1.1–9.0]	**<0.0001**	1.6 (1.5–1.8)
C18:1-PC	22.2 (18.1–25.5) [12.1–34.8]	**<0.0001**	17.0 (15.9–18.0)
C18:2-PC	35.4 (31.9–40.9) [22.1–57.0]	**<0.0001**	49.7 (47.8–52.4)
C20:4-PC	18.9 (17.4–21.3) [12.9–31.6]	**0.0018**	21.1 (19.2–23.2)
C20:5-PC	4.4 (3.1–5.4) [0.7–9.8]	**<0.0001**	1.0 (0.8–1.6)
C22:6-PC	11.9 (10.2–14.0) [3.8–17.9]	**<0.0001**	5.4 (4.6–6.9)

Water-soluble (A) and lipidic (B) parameters of SBS patients. If not otherwise stated, data are presented as medians (interquartile range) and [range] of 80 patients. Abbreviations: C18:1, oleic acid; C18:2, linoleic acid; C20:4, arachidonic acid; C20:5, eicosapentaenoic acid; C22:6, docosahexaenoic acid; DMG, dimethylglycine; PC, phosphatidylcholine; SBS, short bowel syndrome; SPH, sphingomyelin; TMAO, trimethylamine oxide; and *, reference values are from internal routine or study data of healthy adult volunteers and CF patients without exocrine pancreatic insufficiency/without choline deficit. Statistically significant differences are in bold font.

**Table 3:** Patients without or with parenteral nutrition (PN).

	No PN (*n* = 16)	PN (*n* = 64)	*p* Value
A: General Parameters			
Age	7.8 (5.1–11.0) [1.9–13.3]	5.9 (2.1–10.3) [0.2–17.9]	*p* = 0.1780
BMI	14.7 (13.9–15.9) [12.1–23.7]	15.7 (14.4–16.8) [11.4–26.2]	*p* = 0.1054
Conjugated bilirubin (mg/dL)	0.2 (0.1–0.3) [0.1–1.7]	0.2 (0.1–0.4) [0.1–18.4]	*p* = 0.9476
AST (U/L)	28 (25–33) [18–392]	43 (31–55) [18–301]	***p* = 0.0032**
ALT (U/L)	23 (19–27) [11–488]	47 (27–65) [14–275]	***p* = 0.0022**
gGT (U/L)	13 (10–15) [7–427]	24 (14–40) [7–203]	***p* = 0.0012**
AP (U/L)	217 (171–280) [103–597]	296 (219–358) [111–809]	***p* = 0.0082**
Prothrombin time (quick)	96 (89–113) [61–120]	80 (65–88) [31–112]	***p* = 0.0012**
Albumin (g/dL)	3.7 (3.7–3.9) [3.1–4.2]	3.5 (3.2–3.7) [2.0–4.4]	***p* = 0.0286**
Cholesterol (mg/dL)	114 (96–126) [72–220]	98 (74–108) [33–218]	***p* = 0.011**
Triglycerides (mg/dL)	83 (76–136) [35–198]	51 (39–71) [18–232]	***p* = 0.0047**
B: Water-Soluble Choline Metabolites (µmol/L)			
TMAO	3.8 (2.6–8.6) [0.1–29.0]	1.3 (0.1–9.5) [0.0–31.8]	*p* = 0.0877
Choline	8.5 (7.1–10.6) [5.1–12.1]	7.9 (6.2–10.2) [2.5–15.4]	*p* = 0.5936
Betaine	22.8 (16.9–29.4) [8.9–33.0]	19.2 (15.2–24.6) [4.0–193.8]	*p* = 0.4705
Choline + betaine	33.0 (24.1–37.2) [14.0–43.3]	28.9 (22.7–35.7) [6.5–219.0]	*p* = 0.6092
DMG (µmol/L)	3.1 (2.7–4.0) [1.6–6.2]	2.4 (2.1–3.1) [0.8–5.2]	*p* = 0.0201
Carnitine (µmol/L)	22.4 (19.5–24.3) [12.9–31.2]	13.9 (8.7–21.2) [2.1–58.1]	***p* = 0.0018**
C: Phospholipids (mmol/L)			
PC	1.28 (1.21–1.55) [1.08–2.96]	1.23 (1.06–1.47) [0.55–2.32]	*p* = 0.1328
SPH	0.29 (0.27–0.32) [0.21–0.44]	0.27 (0.23–0.31) [0.12–0.46]	*p* = 0.1166
Lyso-PC	0.025 (0.023–0.034) [0.021–0.040]	0.030 (0.024–0.036) [0.006–0.048]	*p* = 0.3799
D: PC Sub-Groups (Mol-% of PC)			
Sat,-PC	1.8 (1.5–2.2) [1.1–2.7]	2.7 (2.4–2.9) [1.7–9.0]	***p* < 0.0001**
C18:1-PC	15.8 (14.2–18.8) [12.1–24.3]	23.2 (20.3–26.5) [14.9–34.8]	***p* < 0.0001**
C18:2-PC	48.3 (42.0–53.4) [38.0–57.0]	33.5 (31.4–36.3) [22.1–47.1]	***p* < 0.0001**
C20:4-PC	22.4 (19.7–23.3) [17.0–31.6]	18.6 (17.2–20.1) [12.9–23.7]	***p* = 0.0007**
C20:5-PC	1.2 (1.0–1.5) [0.7–3.9]	4.8 (3.8–6.0) [1.9–9.8]	***p* < 0.0001**
C22:6-PC	5.4 (4.2–7.0) [3.8–12.2]	12.6 (10.9–14.3) [7.6–17.9]	***p* < 0.0001**

Biometric and serological data (A), water-soluble choline components (B), phospholipids containing a choline headgroup (C), and PC subgroups (D) of SBS patients with or without PN treatment. Data are presented as medians (interquartile range) and [range] of 80 patients. Abbreviations: AST, serum aspartate aminotransferase; ALT, serum alanine aminotransferase; BMI, body mass index; DMG, dimethylglycine; gGT, gamma glutamyl transferase; C18:1, oleic; C18:2, linoleic; C20:4, arachidonic; C20:5, eicosapentaenoic; C22:6, docosahexaenoic; PC, phosphatidylcholine; PN, parenteral nutrition; and TMAO, trimethylamine oxide. Significant differences shown in bold font.

**Table 4:** Patients with or without hepatic steatosis.

	No Steatosis (*n* = 64)	Steatosis (*n* = 16)	*p* Value
A: General Parameters			
Age	5.9 (2.3–10) [0.2–17.9]	8.6 (4.7–12.5) [0.7–16.9]	*p* = 0.1878
BMI	15.5 (14.0–16.5) [11.4–26.2]	15.9) (15.3–16.9) [13.6–23.7]	*p* = 0.1704
RSBL (cm)	45 (25–80) [7–200]	30 (20–55) [6–80]	*p* = 0.2383
PNDI	79 (22–101) [0–160]	106 (67–115) [0–133]	*p* = 0.0810
Parenteral triglycerides (g/kg/d)	0.95 (0.33–1.48) [0.00–2.62]	1.23 (0.94–1.45) [0.00–1.99]	*p* = 0.3389
Parenteral lipid-bound choline (mg/kg/d)	15.8 (5.6–24.7) [0.0–43.7]	20.5 (15.7–24.1) [0.0–33.1]	*p* = 0.3015
Conjugated bilirubin (mg/dL)	0.2 (0.1–0.4) [0.1–18.4]	0.2 (0.1–0.3) [0.1–7.7]	*p* = 0.8065
AST (U/L)	39 (27–49) [18–392]	45 (28–70) [18–301]	*p* = 0.3830
ALT (U/L)	34 (24–56) [11–488]	45 (35–66) [16–275]	*p* = 0.1939
gGT (U/L)	20 (12–34) [7–427]	23 (14–37) [7–47]	*p* = 0.7590
AP (U/L)	276 (203–325) [103–809]	310 (240–336) [155–563]	*p* = 0.3420
Prothrombin time (Quick)	81 (71–95) [31–120]	80 (63–88) [43–97]	*p* = 0.2417
Albumin (g/dL)	3.6 (3.2–3.8) [2.0–4.4]	3.4 (3.2–3.7) [2.2–4.0]	*p* = 0.2468
Cholesterol (mg/dL)	103 (80–117) [45–220]	83 (74–101) [33–123]	*p* = 0.0607
Triglycerides (mg/dL)	57 (39–85) [18–232]	66 (44–73) [32–161]	*p* = 0.7179
B: Water-Soluble Choline Metabolites (µmol/L)			
TMAO	2.6 (0.2–9.0) [0.0–31.8]	2.6 (0.1–14.5) [0.1–24.2]	*p* = 0.8896
Choline	8.5 (7.2–10.6) [2.5–15.4]	5.6 (4.8–8.7) [3.0–10.8]	***p* = 0.0044**
Betaine	21.1 (17.1–25.9) [4.0–193.8]	14.9 (11.8–21.2) [7.6–32.6]	***p* = 0.0257**
Choline + betaine	30.7 (24.5–37.2) [6.5–219.0]	23.8 (16.1–28.3) [10.5–40.4]	***p* = 0.0085**
DMG	2.6 (2.2–3.4) [0.8–6.2]	2.2 (2.0–3.0) [1.0–3.8]	*p* = 0.1307
Carnitine	14.8 (9.9–22.5) [2.1–49.6]	19.5 (12.9–23.4) [7.3–58.1]	*p* = 0.2002
C: Phospholipids (mmol/L)			
PC	1.27 (1.13–1.53) [0.55–2.96]	1.16 (1.02–1.36) [0.83–1.64]	*p* = 0.1058
SPH	0.28 (0.24–0.31) [0.12–0.45]	0.27 (0.23–0.29) [0.19–0.34]	*p* = 0.2177
Lyso-PC	0.031 (0.023–0.036) [0.056–0.048]	0.025 (0.020–0.028) [0.011–0.042]	***p* = 0.0230**
D: PC Sub-Groups (Mol-% of PC)			
Sat, -PC	2.5 (2.3–2.8) [1.1–9.0]	2.7 (2.3–2.9) [1.5–7.9]	*p* = 0.3406
C18:1-PC	22.2 (17.8–25.5) [12.1–34.8]	22.8 (18.7–25.4) [14.2–33.3]	*p* = 0.9090
C18:2-PC	35.4 (31.9–41.3) [22.3–57.0]	35.0 (32.6–38.6) [22.1–45.9]	*p* = 0.8006
C20:4-PC	19.0 (17.4–21.3) [14.2–31.6]	18.4 (17.5–20.8) [12.9–24.1]	*p* = 0.5356
C20:5-PC	4.3 (2.5–5.4) [0.7–9.8]	4.7 (3.5–5.5) [1.6–7.0]	*p* = 0.4378
C22:6-PC	11.6 (9.7–14.0) [3.8–17.9]	12.6 (11.1–14.0) [7.3–16.9]	*p* = 0.2659

Biometric and serological data (A), water-soluble choline components (B), phospholipids containing a choline headgroup (C), and PC subgroups (D) of SBS patients with or without hepatic steatosis. Data are presented as medians (interquartile range) and [range] of 80 patients. Abbreviations: AST, serum aspartate aminotransferase; ALT, serum alanine aminotransferase; BMI, body mass index; DMG, dimethylglycine; gGT, gamma glutamyl transferase; C18:1, oleic; C18:2, linoleic; C20:4, arachidonic; C20:5, eicosapentaenoic; C22:6, docosahexaenoic; PC, phosphatidylcholine; and TMAO, trimethylamine oxide. Significant differences in bold.

## Data Availability

The original contributions presented in this study are included in the article/Supplementary Material. Further inquiries can be directed to the corresponding author.

## Supplementary Materials

The following supporting information can be downloaded at: https://www.mdpi.com/article/10.3390/nu18101553/s1. Table S1: Characteristics of SBS sub-group patients; Table S2: Choline and choline-related compounds in SBS sub-group patients; Table S3: Presence or absence of the ileocecal valve. Figure S1. (**A**): Age distribution of SBS patients with and without steatosis in rela-tion to parenterally administered PC with and without steatosis. 1–<6 y: no steatosis, *n* = 14; steatosis, *n* = 5; 6–<12 y: no steatosis, *n* = 18; steatosis, *n* = 5; 12–<18 y: no steatosis, *n* = 32; steatosis, *n* = 6. (B–D) show the amounts of intravenous fat, PC and choline equivalent relative to sex and the absence of presence of steatosis of the liver.

## Abbreviations

The following abbreviations are used in this manuscript:

AI	adequate intake
ARA/C20:4	arachidonic acid
BMI	body mass index
DHA/C22:6	docosahexaenoic acid
DMG	dimethylglycine
EPA/C20:5	eicosapentaenoic acid
FIP	focal intestinal perforation
GSH	glutathione
IF	intestinal failure
IFALD	intestinal failure-associated liver disease
LA/C18:2	linoleic acid
LC-PUFA	long-chain polyunsaturated fatty acid
OA/C18:1	oleic acid
PC	phosphatidylcholine
PEMT	phosphatidylethanolamine N-methyl transferase
PN	parenteral nutrition
sPLA2IB	pancreatic phospholipase A2 IB
SBS	short bowel syndrome
SIBO	small intestinal bacterial overgrowth
SNPs	Single-nucleotide polymorphisms
SPH	sphingomyelin
VLDLs	very-low-density lipoproteins
