# Supplementary material for: Choline and Choline-Related Metabolites in Pediatric Short Bowel Syndrome
Source: Nutrients. 2026 May 14;18(10):1553. doi: 10.3390/nu18101553 (PMC13209643; doi:10.3390/nu18101553)
Supplement: Supplementary file 1 [file nutrients-18-01553-s001.zip › nutrients-4242659-supplementary.pdf]

Table S1: Characteristics of SBS sub-group patients

SBS Background	NEC (31)	Gastroschisis (13)	Volvulus (13)	Atresia (11)	Other (12)
Age (y)	5.7 (2.6-9.7) [0.2-17.9]	6.4 (1.8-9.0) 0.8-15.0	9.2 (3.4-14.8) 1.3-16.8	7.2(3.2-11.6) 0.3-13.6	4.8 (2.3-9.4) 0.9-14.2
Sex (male/female)	16/15	4/9	12/1	4/7	8/4
Body weight (kg)	17.2 (10.4-26.2) [3.6-65.5]	19.0 (10.7-22.0) [6.5-51.2]	26.5 (15.6-46.6) [9.3-52.8]	19.1 (10.7-25.6) [3.8-34.1]	15.7 (11.7-23.7) [6.9-46.5]
Body length (cm)	111.5 (86.8-128.8) [50.5-160.0]	114.8 (79.0-118.0) [65.0-167.0]	128.0 (100.8-159.5) [75.0-169.0]	118.0 (85.1-128.8) [58.0-146.5]	104.7 86.3-126.9) [66.0-159.8]
BMI (kg/m^2)	15.5 (14.0-16.4) [12.1-26.2]	15.4 (14.4-17.1) [11.9-19.2]	16.4 (16.0-18.3) [14.4-18.5]	14.7 (13.6-15.8) [11.4-16.2]	15.7 (14.5-16.6) [13.0-18.3]
Parenteral Nutrition (No/Yes)	10/21	0/13	1/12	3/8	2/10
Formula (No/Yes/Unknown)	16/14/1	6/7/0	3/10	4/5/2	1/10/1
Terminal Ileum Present (No/Yes)	21/10	12/1	5/8	5/6	7/5
Stoma (No/Yes)	27/4	10/3	13/0	10/1	8/4
ICV present (No/Yes/Unknown)	24/6/1	13/0/0	5/8/0	7/4/0	10/2/0
RSBL (cm)	43 (21-63) [0-140]	43 (24-59) [7-140]	20 (7-35) [0-120]	45 (33-3) [15-100]	70 (20-88) [0-200]
PNDI (No/Yes	68 (0-98) [0-160]	95 (88-107) [79-133]	90 (64-108) [0-127]	84 (10-110) [0-124]	61 (43-108) [0-129]
Hepatomegaly (No/Yes)	24/7	11/2	12/1	10/1	9/3
Steatosis (No/Yes)	24/7	9/4	12/1	9/2	10/2
Albumin (g/dL) [3.0-5.0]	3.5 (3.3-3.8) [2.2-4.4]	3.4 (3.1-3.5) [2.7-4.1]	3.6 (3.5-4.0) [3.0-4.2]	3.7 (3.6-3.9) [2.9-4.0]	3.4 (3.0-3.7) [2.0-4.1]
Prothrombin time (Quick) [70-120]	85 (79-91) [31-120]	72 (60-89) [47-109]	74 (65-93) [33-97]	86 (69-95) [59-116]	80 (75-93) [51-105]
C-reactive Protein (mg/L)	0.05 (0.04-0.12) [0.01-0.98]	0.64 (0.05-1.15) [0.03-5.74]	0.05 (0.03-0.05) [0.00-0.14]	0.05 (0.01-0.13) [0.011.08]	0.10 (0.05-0.46) [0.012.46]
Cholesterol (mg/dL) [130-190]	1035 (85-114) [49-153]	82 (68-106) [33-129]	74 (62-103) [49-113]	102 (89-116) [74-220]	106 (89-137) [45-218]
Triglycerides (mg/dL) [<200]	65 (44-75) [18-161]	47 (44-80) [31-117]	40 (36-46) [25-105]	70 (52-127) [32-198]	74 (47-152) [27-232]
Phosphatidylcholine (µmol/L)	1.27 (1.16-1.39) [1.03-2.00]	1.17 (1.00-1.64) [0.74-1.76]	0.98 (0.79-1.29) [0.55-1.55]	1.23 (1.04-1.71) [0.83-2.96]	1.43 (1.22-1.65) [1.15-2.32]
AST [<39 U/L]	40 (28-55) [18-301]	45 (35-57) [21-97]	33 (27-54) [20-105]	36 (27-41) [18-392]	41 (28-47) [21-110]
ALT [<39U/L]	44 (25-62) [11-275]	39 (335-56) [14-112]	31 (27-47) [20-263]	34 (28-53) [21-488]	39 (18-54) [14-256]
gGT [<30U/L]	20 (12-41) [7-203]	23 (19-31) [8-51]	16 (14-29) [11-59]	16 (11-34) [7-427]	21 (12-38) [8-88]

Biometric and serological data of the SBS subgroups. Data are medians, (interquartile ranges) and [range] of indicated patient numbers. Abbreviations: AST, serum aspartate aminotransferase; ALT, serum alanine aminotransferase; BMI, body mass index; FIP, focal intestinal perforation; gGT, gamma glutamyl transferase; ICV, ileocecal valve; NEC, necrotizing enterocolitis; PNDI, parenteral nutrition dependency index; RSBL, residual short bowel length; SBS, short bowel syndrome; SIBO, small intestinal bacterial overgrowth.

Table S2: Choline and choline-related compounds in SBS sub-group patients

SBS Background	NEC (31)	Gastroschisis (13)	Volvulus (13)	Atresia (11)	Other (12)
Age (y)	5.7 (2.6-9.7) [0.2-17.9]	6.4 (1.8-9.0) 0.8-15.0	9.2 (3.4-14.8) 1.3-16.8	7.2(3.2-11.6) 0.3-13.6	4.8 (2.3-9.4) 0.9-14.2
Sex (male/female)	16/15	4/9	12/1	4/7	8/4
Body weight (kg)	17.2 (10.4-26.2) [3.6-65.5]	19.0 (10.7-22.0) [6.5-51.2]	26.5 (15.6-46.6) [9.3-52.8]	19.1 (10.7-25.6) [3.8-34.1]	15.7 (11.7-23.7) [6.9-46.5]
Body length (cm)	111.5 (86.8-128.8) [50.5-160.0]	114.8 (79.0-118.0) [65.0-167.0]	128.0 (100.8-159.5) [75.0-169.0]	118.0 (85.1-128.8) [58.0-146.5]	104.7 86.3-126.9) [66.0-159.8]
BMI (kg/m^2)	15.5 (14.0-16.4) [12.1-26.2]	15.4 (14.4-17.1) [11.9-19.2]	16.4 (16.0-18.3) [14.4-18.5]	14.7 (13.6-15.8) [11.4-16.2]	15.7 (14.5-16.6) [13.0-18.3]
Parenteral Nutrition (No/Yes)	10/21	0/13	1/12	3/8	2/10
Formula (No/Yes/Unknown)	16/14/1	6/7/0	3/10	4/5/2	1/10/1
Terminal Ileum Present (No/Yes)	21/10	12/1	5/8	5/6	7/5
Stoma (No/Yes)	27/4	10/3	13/0	10/1	8/4
ICV present (No/Yes/Unknown)	24/6/1	13/0/0	5/8/0	7/4/0	10/2/0
SIBO (No/Yes/Unclear)	4/13/14	0/10/3	2/3/8	3/4/4	6/3/3
RSBL (cm)	43 (21-63) [0-140]	43 (24-59) [7-140]	20 (7-35) [0-120]	45 (33-3) [15-100]	70 (20-88) [0-200]
PNDI (No/Yes	68 (0-98) [0-160]	95 (88-107) [79-133]	90 (64-108) [0-127]	84 (10-110) [0-124]	61 (43-108) [0-129]
Hepatomegaly (No/Yes)	24/7	11/2	12/1	10/1	9/3
Steatosis (No/Yes)	24/7	9/4	12/1	9/2	10/2
Albumin (3.0-5.0 g/dL)	3.5 (3.3-3.8) [2.2-4.4]	3.4 (3.1-3.5) [2.7-4.1]	3.6 (3.5-4.0) [3.0-4.2]	3.7 (3.6-3.9) [2.9-4.0]	3.4 (3.0-3.7) [2.0-4.1]
Prothrombin time (Quick) [70-120]	85 (79-91) [31-120]	72 (60-89) [47-109]	74 (65-93) [33-97]	86 (69-95) [59-116]	80 (75-93) [51-105]
C-reactive Protein (mg/L)	0.05 (0.04-0.12) [0.01-0.98]	0.64 (0.05-1.15) [0.03-5.74]	0.05 (0.03-0.05) [0.00-0.14]	0.05 (0.01-0.13) [0.011.08]	0.10 (0.05-0.46) [0.012.46]
Cholesterol (130-190 mg/dL)	1035 (85-114) [49-153]	82 (68-106) [33-129]	74 (62-103 [49-113]	102 (89-116) [74-220]	106 (89-137) [45-218]
Triglycerides (<200 mg/dL)	65 (44-75) [18-161]	47 (44-80) [31-117]	40 (36-46) [25-105]	70 (52-127) [32-198]	74 (47-152) [27-232]
Phosphatidylcholine (1.71[1.55-1.88] mmol/L)	1.27 (1.16-1.39) [1.03-2.00]	1.17 (1.00-1.64) [0.74-1.76]	0.98 (0.79-1.29) [0.55-1.55]	1.23 (1.04-1.71) [0.83-2.96]	1.43 (1.22-1.65) [1.15-2.32]

AST [<39 U/L]	40 (28-55) [18-301]	45 (35-57) [21-97]	33 (27-54) [20-105]	36 (27-41) [18-392]	41 (28-47) [21-110]
ALT [<39U/L]	44 (25-62) [11-275]	39 (335-56) [14-112]	31 (27-47) [20-263]	34 (28-53) [21-488]	39 (18-54) [14-256]
gGT [<30U/L]	20 (12-41) [7-203]	23 (19-31) [8-51]	16 (14-29) [11-59]	16 (11-34) [7-427]	21 (12-38) [8-88]

Biometric and serological data of the SBS subgroups. Data are medians, (interquartile ranges) and [range] of indicated patient numbers. Abbreviations: AST, serum aspartate aminotransferase; ALT, serum alanine aminotransferase; BMI, body mass index; FIP, focal intestinal perforation; gGT, gamma glutamyl transferase; ICV, ileocecal valve; NEC, necrotizing enterocolitis; PNDI, parenteral nutrition dependency index; RSBL, residual short bowel length; SBS, short bowel syndrome; SIBO, small intestinal bacterial overgrowth.

Table S3. Presence or absence of the ileocecal valve

	Intact (n=20)	Not intact (n=60)	
PN (Yes/No)	14/6	50/10	
Parenteral Triglycerides (g/kg/d)	0.78 (0.09-1.04) [0.00-2.16]	1.14 (0.57-1.49) [0.00-2.62]	p= 0.1221
Parenteral lipid-bound choline as egg-PC (mg/kg/d)	12.9 (1.4-17.3) [0.0-36.0]	19.0 (9.5-24.8) [0.0-43.7]	**p< 0.0001**
A: General Parameters			
Age	5.6 (2.0-9.3) [0.3-15.4]	6.5 (2.4-10.9) [0.2-17.9]	p= 0.5976
BMI	15.0 (14.3-16.7) [11.4-18.5]	15.7 (14.4-16.4) [11.9-26.2]	p= 0.6592
Conjugated Bilirubin (mg/dL)	0.2 (0.1-0.4) [0.1-4.8]	0.2 (0.1-0.3) [0.1-18.4]	p= 0.4145
AST (U/L)	28 (25-37) [20-392]	43 (31-54) [18-301]	**p= 0.0156**
ALT (U/L)	27 (22-42) [14-488]	45 (25-65) [11-275]	p= 0.1281
gGT (U/L)	15 (12-19) [10-427]	25 (13-39) [7-203]	p= 0.1580
AP (U/L)	295 (212-424) [111-620]	278 (204-318) [103-809]	p= 0.4367
Prothrombin time (Quick)	80 (65-89) [33-97]	81 (70-95) [31-120]	p= 0.5052
Albumin (g/dL)	3.8 (3.6-4.0) [3.3-4.2]	3.4 (3.1-3.7) [2.0-4.4]	**p= 0.0004**
Cholesterol (mg/dL)	99 (71-114) [49-220]	101 (79-113) [33-218]	p= 0.9894
Triglycerides (mg/dL)	46 (35-71) [25-142]	66 (44-84) [18-232]	p= 0.1142
B: Water-Soluble Choline Metabolites (µmol/L)			
TMAO (µmol/L)	5.25 (1.10-18.30) [0.08-31.84]	1.2 (0.1-7.4) [0.0-29.0]	**p=0.0167**
Choline (µmol/L)	7.8 (6.8-10.6) [4.6-12.8]	8.4 (6.5-10.7) [2.5-33.3]	p=0.3498
Betaine (µmol/L)	22.1 (17.5-25.7) [8.9-42.9]	19.6 (15.0-25.1) [4.0-193.8]	p= 0.4269
Choline+Betaine	31.3 (24.4-35.5) [14.0-53.7]	28.4 (22.6-37.7 [6.5-219]	p= 0.6729
DMG (µmol/L)	2.5 (2.2-3.7) [0.8-4.3]	2.5 (2.1-3.1) [1.0-6.2]	p= 0.8587
Carnitine (µmol/L)	21.8 (9.3-23.8) [2.1-38.5]	15.2 (10.4-21.5) [3.7-58.1]	p= 0.5485
C: Phospholipids (mmol/L)			
PC (mmol/L)	1.27 (0.98-1.57) [0.55-2.96]	1.24 (1.12-1.47) [0.74-2.32]	p= 0.8720
SPH (mmol/L)	0.30 (0.22-0.35) [0.16-0.44]	0.27 (0.24-0.30) [0.12-0.45]	p= 0.1518
Lyso-PC (mmol/L)	0.033 (0.023-0.037) [0.017-0.045]	0.029 (0.023-0.035) [0.006-0.048]	p= 0.4634
D: PC Sub-groups (Mol-% of PC)			
Sat,-PC (mol-%)	2.6 (2.4-2.7) [1.4-4.7]	2.6 (2.3-2.9) [1.1-9.0]	p= 0.8020
C18:1-PC (mol-%)	21.1 (16.3-22.8) [13.5-28.8]	22.8 (18.4-26.1) [12.1-34.8]	p= 0.1252
C18:2-PC (mol-%)	37.1 (33.2-42.0) [24.1-55.2]	34.6 (31.6-39.5) [22.1-57.0]	p= 0.1471
C20:4-PC (mol-%)	20.4 (19.1-21.6) [14.2-31.6]	18.1 (17.2-20.7) [12.9-28.8]	**p= 0.0117**
C20:5-PC (mol-%)	3.9 (2.1-5.6) [0.9-9.8]	4.4 (3.4-5.4) [0.7-9.0]	p= 0.5337
C22:6-PC (mol-%)	10.7 (7.4-12.3) [3.9-15.6]	12.4 (10.4-14.3) [3.8-17.9]	**p= 0.0393**

Biometric and serological data (A), water-soluble choline components (B), phospholipids containing a choline headgroup (C), and PC subgroups (D) of SBS patients with or without an intact ileocecal valve, irrespective of PN treatment. Data are medians (interquartile) and [range] of 80 patients. Abbreviations: AST, serum aspartate aminotransferase; ALT, serum alanine aminotransferase; BMI, body mass index; DMG, dimethylglycine; gGT, gamma glutamyl transferase; C18:1, oleic; C18:2, linoleic; C20:4, arachidonic; C20:5, eicosapentaenoic; C22:6,

docosahexaenoic; PC, phosphatidylcholine; TMAO, trimethylamine oxide. Significant differences shown in bold font.

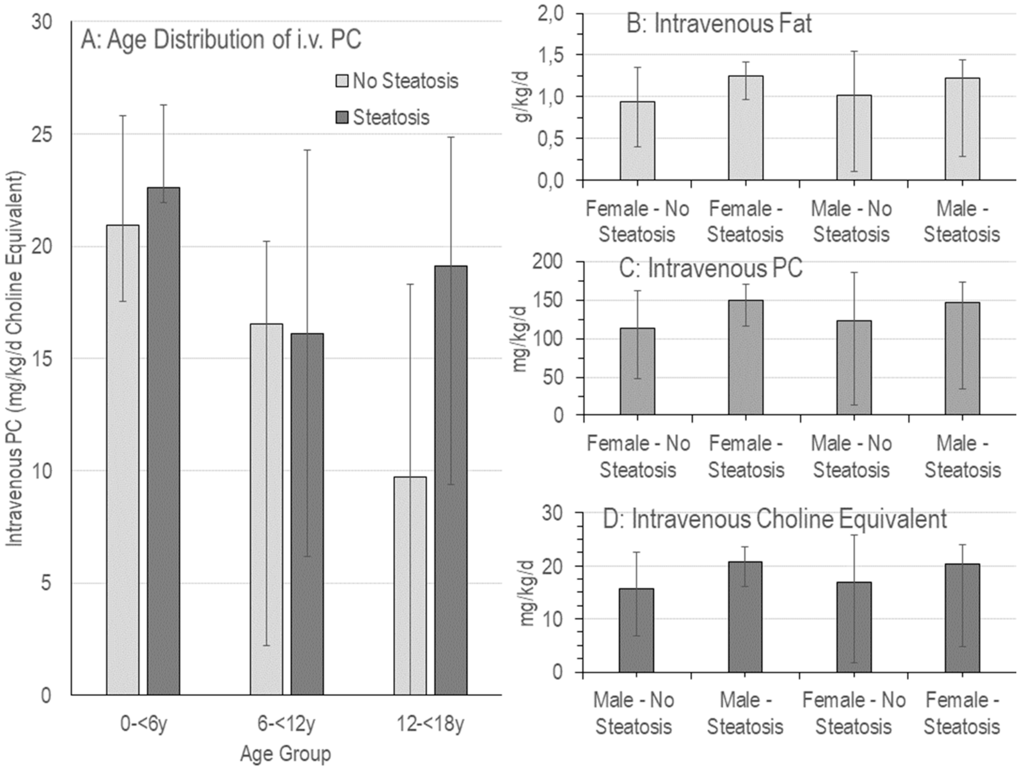

Supplementary Figure S1. A: Age distribution of SBS patients with and without steatosis in rela-tion to parenterally administered PC with and without steatosis. 1-<6y: no steatosis, N=14; stea-tosis, N=5; 6-<12y: no steatosis, N=18; steatosis, N=5; 12-<18y: no steatosis, N=32; steatosis, N=6. B-D show the amounts of intravenous fat, PC and choline equivalent relative to sex and the ab-sence of presence of steatosis of the liver.
